# Experimental Verification of the Balance between Elastic Pressure and Ionic Osmotic Pressure of Highly Swollen Charged Gels

**DOI:** 10.3390/gels7020039

**Published:** 2021-04-01

**Authors:** Tasuku Nakajima, Ken-ichi Hoshino, Honglei Guo, Takayuki Kurokawa, Jian Ping Gong

**Affiliations:** 1 Faculty of Advanced Life Science, Hokkaido University, Sapporo 001-0021, Japan; ken-ichi_hoshino@sci.hokudai.ac.jp (K.-i.H.); hlguo@sci.hokudai.ac.jp (H.G.); kurokawa@sci.hokudai.ac.jp (T.K.); gong@sci.hokudai.ac.jp (J.P.G.); 2 Global Station for Soft Matter, Hokkaido University, Sapporo 060-0808, Japan; 3 Institute for Chemical Reaction Design and Discovery (WPI-ICReDD), Hokkaido University, Sapporo 001-0021, Japan

**Keywords:** swelling, elasticity, osmotic pressure, Donnan potential, tetra-PEG gel, molecular stent method

## Abstract

The equilibrium swelling degree of a highly swollen charged gel has been thought to be determined by the balance between its elastic pressure and ionic osmotic pressure. However, the full experimental verification of this balance has not previously been conducted. In this study, we verified the balance between the elastic pressure and ionic osmotic pressure of charged gels using purely experimental methods. We used tetra-PEG gels created using the molecular stent method (St-tetra-PEG gels) as the highly swollen charged gels to precisely and separately control their network structure and charge density. The elastic pressure of the gels was measured through the indentation test, whereas the ionic osmotic pressure was determined by electric potential measurement without any strong assumptions or fittings. We confirmed that the two experimentally determined pressures of the St-tetra-PEG gels were well balanced at their swelling equilibrium, suggesting the validity of the aforementioned relationship. Furthermore, from single-strand level analysis, we investigated the structural requirements of the highly swollen charged gels in which the elasticity and ionic osmosis are balanced at their swelling equilibrium.

## 1. Introduction

Charged gels, which have fixed charges on their polymer chains, exhibit a large degree of equilibrium swelling in polar solvents and possess an environmentally responsive swelling ability [1,2,3,4,5]. Therefore, charged gels have attracted great attention as superabsorbents, soft actuators, and drug carriers for drug delivery systems [6,7,8,9,10]. As these charged gels have such a wide range of possible applications, it is desirable to tune the equilibrium swelling degree and mechanical properties of these gels by altering the charge density, bath solution, network structure, etc. Therefore, experimental and theoretical approaches to understand the swelling mechanism of charged gels are extremely useful, and numerous studies have been conducted to date.

Generally, the equilibrium swelling state of a gel has been considered to be determined by the balance between its elastic pressure, $\Pi_{\mathrm{el}}$, and its osmotic pressure, $\Pi_{\mathrm{os}}$ [11,12,13,14,15]. The origin of the elastic pressure of a common gel is the elastic energy of its polymer network. When a gel absorbs solvent molecules and swells, its network strands are stretched, and consequently, elastic energy is accumulated in the network [11]. On the other hand, the osmotic pressure of a charged gel is separable into two terms. One term, called the osmotic pressure of mixing, $\Pi_{\mathrm{mix}}$, is based on the free energy of mixing a polymer and a solvent, which is generally explained by the classical Flory–Huggins theory [13,14,15]. The other term, called ionic osmotic pressure, $\Pi_{\mathrm{ion}}$, is based on the difference in the concentration of mobile ions inside and outside the gel, which is specific to charged gels [14,16,17,18]. The other pressures of a charged gel, such as the pressure originating from electrostatic repulsion between the fixed ions, were also taken into account in the rigorous modeling of a charged gel, following some previous papers, which we assumed that such additional terms are excessively small to be ignorable relative to $\Pi_{\mathrm{ion}}$ [16,17]. As the pressure of a charged gel should be balanced at its equilibrium swelling state, the equilibrium swelling state of charged gels can be expressed by Equation (1):

(1) $$\Pi_{\mathrm{el}}+\Pi_{\mathrm{mix}}+\Pi_{\mathrm{ion}}=0$$

$\Pi_{\mathrm{el}}$ is generally equal to its shear modulus, *G* [19,20], and can be determined from mechanical tests:

(2) $$\Pi_{\mathrm{el}}=-G$$

$\Pi_{\mathrm{mix}}$ follows the scaling relationship for the osmotic pressure of semidilute polymer solutions [21]:

(3) $$\Pi_{\mathrm{mix}}=K\phi^{\frac{3\upsilon }{3\upsilon -1}},$$

where ϕ is the polymer volume fraction, *K* is a parameter that depends on various factors such as the gel preparation conditions, and υ is the Flory exponent, which depends on the polymer–solvent affinity (0.5 for theta solvent and 0.588 for the athermal solvent). $\Pi_{\mathrm{ion}}$ originates from the difference in the activity of the total mobile ions between the gel and the bath solution, $\Delta \alpha_{\mathrm{ion}}$, and is given by

(4) $$\Pi_{\mathrm{ion}}=kT\sum_{i}\left(\alpha_{i}^{g}-\alpha_{i}^{env}\right)=\Delta \alpha_{\mathrm{ion}}kT,$$

where *k* is the Boltzmann constant, *T* is the absolute temperature, and α is the activity of the ion in the subsystem [16]. For α, the subscripts and superscripts denote the ion species and subsystem (*g* is the gel and *env* is the bath solution), respectively.

Equation (1) can be simplified in the case of highly swollen charged gels. A charged gel swells excessively in polar solvents of low salt concentration if it contains dense dissociatable ionic groups, owing to the large $\Pi_{\mathrm{ion}}$ originating from dense mobile counterions inside the gels. As the large swelling of such gels lowers their polymer volume fraction ϕ and corresponding $\Pi_{\mathrm{mix}}$, the contribution of $\Pi_{\mathrm{mix}}$ to the total osmotic pressure of highly swollen charged gels becomes negligible, as suggested by theoretical considerations [22,23,24] and calculations [20,25]. Thus, the equilibrium swelling state of highly swollen charged gels should be approximately determined by the balance between the elastic pressure and ionic osmotic pressure, as shown in Equation (5):

(5) $$-\Pi_{\mathrm{el}}\approx \Pi_{\mathrm{ion}}$$

The substitution of Equations (2) and (4) into Equation (5) obtains:

(6) $$G\approx \Delta \alpha_{\mathrm{ion}}kT.$$

**Figure 1:**
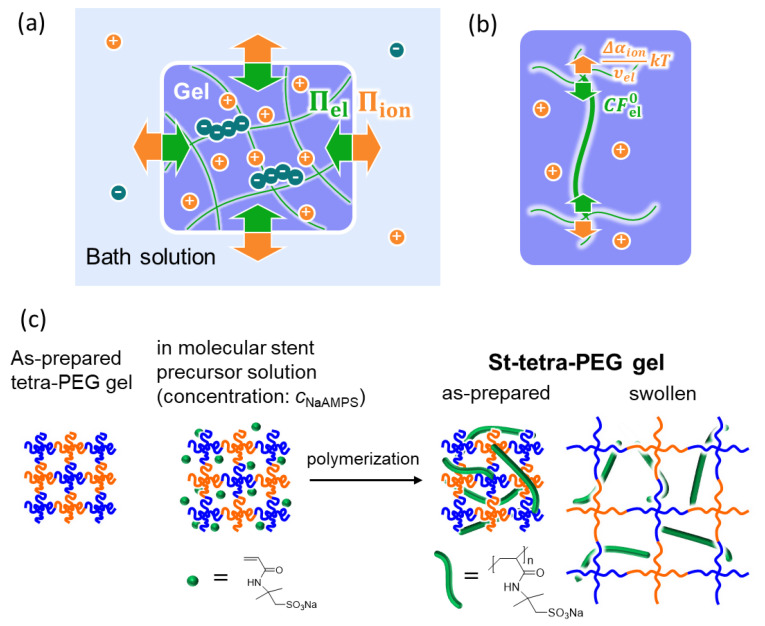
(**a**) Schematic illustration of the balance between the elastic pressure (symbolically expressed by green arrows) and ionic osmotic pressure (orange arrows) of a highly swollen charged gel at swelling equilibrium, where + and – are cations and anions, respectively; (**b**) balance between elasticity and ionic osmosis at a single-strand level; and (**c**) the synthesis pathway of the St-tetra-PEG gels used in this work.

Equations (5) and (6) describe the swelling equilibrium state of the highly swollen charged gels. Figure 1a shows a schematic of these equations.

The relationship described by Equation (6) is related to the network structure of highly swollen charged gels. According to the molecular-based approaches to determine the elasticity of gels, the shear modulus of a gel is the sum of the elastic energy of each network strand of the gel. By using the average elastic energy of a network strand, $F_{\mathrm{el}}^{0}$, *G* of the gel can be expressed as

(7) $$\Pi_{\mathrm{el}}=-G=-C\upsilon_{\mathrm{el}}F_{\mathrm{el}}^{0},$$

where $\upsilon_{\mathrm{el}}$ is the number density of elastically effective network strands in the gel, *C* is a pre-factor taking a value between *f*_c_-2/*f*_c_ and 1, which represents the fluctuation of the cross-linking points, and *f*_c_ is the functionality of the cross-linking points [26]. Combining Equations (6) and (7) leads to the relationship between the elastic energy per strand (considering the fluctuation of the cross-linking points) and the ionic osmotic pressure of highly swollen charged gels as follows:

(8) $$CF_{\mathrm{el}}^{0}\approx \frac{\Delta \alpha_{\mathrm{ion}}}{\upsilon_{\mathrm{el}}}kT.$$

Equation (8) denotes the balance between elasticity and the ionic osmosis of a highly swollen charged gel at the single-strand level. Figure 1b shows a schematic of this equation. The right side of Equation (8) is the ionic osmotic pressure of the gel divided by the effective network strand density, which corresponds to the average stretching energy applied to each effective strand. The left side of Equation (8) is the average elastic energy borne by each effective strand. At the swelling equilibrium, the two energies should be balanced.

Equations (6) and (8) have quite simple forms compared to the general equations of state equilibrium swelling of gels, although their applicability should be limited to the high swelling regime. The validity of Equation (6) or similar relationships has been suggested by some previous experimental and theoretical works [4,16,22,24,27,28,29]. However, the completely experimental validation of these equations has rarely been reported, especially for Equation (8), due to the following three difficulties. The first difficulty was with the direct experimental determination of ionic activities, which makes it difficult to determine $\Delta \alpha_{\mathrm{ion}}$ and $\Pi_{\mathrm{ion}}$. In many experimental studies, $\Delta \alpha_{\mathrm{ion}}$ and the corresponding $\Pi_{\mathrm{ion}}$ are not directly determined by experiments but are theoretically calculated considering Donnan equilibrium and Manning’s counterion condensation theory [30]. However, because this calculation process requires assumptions or fittings for some parameters, it is hard to say that $\Pi_{\mathrm{ion}}$ has been completely experimentally determined using these methods. In contrast, several reports have attempted to estimate ionic osmotic pressure through measuring electric potential [29,31,32]. For example, Utz et al. measured the Donnan potential of the charged gel, which corresponds to the experimental determination of $\Delta \alpha_{\mathrm{ion}}$, and reported that the obtained swelling and elastic properties of the charged gels were consistent with their Donnan potential to some extent [29]. Unfortunately, their research has been limited to qualitative discussions. The second difficulty is the random and highly inhomogeneous network structure of gels, which makes it difficult to determine $\upsilon_{\mathrm{el}}$ [33,34]. Although the $\upsilon_{\mathrm{el}}$ of rubbery materials has been theoretically estimated from their modulus using the assumption that the network strands follow Gaussian statistics [14,15], the reliability of such indirect information remains questionable. The third difficulty is in controlling the network structure and ion concentrations independently. For the verification of the effect of ionic osmotic pressure on gel swelling, it is desirable to synthesize a series of gels having a constant network structure but different fixed polyion densities. One of the easiest ways to achieve this is to co-polymerize ionic and non-ionic monomers having the same total concentration but different mixing ratios in the presence of cross-linkers [35]. However, even if the total monomer and cross-linker concentrations are constant, the network structure changes depending on the monomer mixing ratio due to the different reactivity ratios of the monomers and the cross-linker for the co-polymerization. Thus, the independent control of the ion concentrations and network structure of charged gels is complicated by using this method. Another conventional method involves controlling the dissociation of counterions by controlling the pH of the bath solution [36]. However, because the high ionic strength of the bath solutions at these pH levels suppresses swelling, charged gels do not swell as much in these pH-controlled bath solutions.

The purpose of this study was to overcome these three difficulties and to verify Equations (6) and (8), which describe the balance between the elasticity and ionic osmosis of a highly swollen charged gel at the macroscale and single-strand scale, using fully experimental methods. First, following previous reports, the microelectrode technique was used to determine the ionic osmotic pressure. In this method, a charged gel equilibrated in a salt solution was prepared, and the electric potential difference between the gel and the bath solution was determined by inserting a reference electrode into the solution and a working electrode into the gel. The obtained potential difference is equal to the Donnan potential of the gel if the concentration of the salt solution is sufficiently low and the working electrode is thin enough [32]. $\Delta \alpha_{\mathrm{ion}}$ and the corresponding $\Pi_{\mathrm{ion}}$ can be determined from the obtained Donnan potential. Second, tetra-PEG gels were used to determine $\upsilon_{\mathrm{el}}$, where PEG stands for polyethylene glycol. Tetra-PEG gels are known as gels with well-defined structures, which are synthesized by an end cross-coupling reaction between the two reactive four-armed prepolymers with controlled molecular weights [37,38]. Owing to the network regularity, the $\upsilon_{\mathrm{el}}$ of tetra-PEG gels has been determined based on the conversion ratio of the end cross-coupling reaction, which can be directly clarified by spectroscopic methods [39,40]. The obtained $\upsilon_{\mathrm{el}}$ of the tetra-PEG gel corresponds well with its elastic properties, suggesting the high reliability of the determined $\upsilon_{\mathrm{el}}$ [41,42]. The tetra-PEG-like system has been used to investigate the swelling mechanism of a moderately swollen charged gel [21]. Third, the molecular stent method was adopted for the independent control of the network structure and polyion density in the gel. In this method, an original gel is first synthesized, and linear polyelectrolyte chains (molecular stents) are then introduced into the original gel network [43]. The long linear polyelectrolyte chains are trapped in the tetra-PEG network within a certain experimental time. The gel incorporated with the molecular stent, called St-gel, greatly swells in polar solvents because of the large ionic osmotic pressure originating from the dissociated counterions of the molecular stent. The elastic pressure of a St-gel is borne by the tetra-PEG network, whereas its ionic osmotic pressure mainly originates from the counterions of the molecular stent. Thus, elasticity and ionic osmosis can be controlled separately. Moreover, if a St-gel is made from a nonionic network, there is no fixed charge on its network strands. Therefore, the effect of the interactions between fixed charges on the swelling of a St-gel should be negligible, which simplifies the gel swelling problem. The molecular stent method has already been used for tetra-PEG gels [26,44], and the relationship between elasticity and swelling degree was investigated.

The following experiments were performed. First, the tetra-PEG gels were prepared as the original materials (reference state). Subsequently, tetra-PEG gels containing molecular stents with various concentrations (St-tetra-PEG gels) were prepared and immersed in 10^−4^ M NaCl aqueous solution until the swelling equilibrium (state of interest) was reached. The synthesis pathway of the St-tetra-PEG gels is illustrated in Figure 1c. The swelling ratio, *Q*, was determined from the volume of the gel at the reference state and at the state of interest. The $\upsilon_{\mathrm{el}}$ of the St-tetra-PEG gels was determined from the preparation condition, reaction conversion, and *Q*. The shear modulus, *G*, of each St-tetra-PEG gel, was determined by the indentation test. The potential difference, Δϕ, was determined using the microelectrode technique, and $\Delta \alpha_{\mathrm{ion}}$ was calculated from Δϕ. The validity of Equations (6) and (8) was directly confirmed using the obtained experimental parameters without any strong assumptions or fitting parameters.

## 2. Results and Discussion

### 2.1. Swelling Ratio and Modulus of the St-tetra-PEG Gels

We synthesized tetra-PEG gel from prepolymer solutions at overlapping concentrations. The as-prepared tetra-PEG gel had a shear modulus *G* of 6.35 ± 0.40 kPa. In addition, the $\upsilon_{\mathrm{el}}$ of the as-prepared tetra-PEG gel of the same composition was estimated to be 1.97 × 10^24^ m^−3^ based on the tree-like theory with the reaction efficiency of the cross-end coupling determined by infrared spectroscopy [26,40]. Thus, $CF_{\mathrm{el}}^{0}$, the elastic energy per strand considering the fluctuation of the cross-linking points of the as-prepared tetra-PEG gel was calculated as $CF_{\mathrm{el}}^{0}=G/\upsilon_{\mathrm{el}}=$ 3.22 × 10^−21^ J. We think that this value is reasonable because it is on the order of *kT* (4.11 × 10^−21^ J at 298 K). Then, the linear poly(2-acrylamido-2-methylpropanesulfonic acid sodium salt (PNaAMPS) of various concentrations, working as a molecular stent, were synthesized in the tetra-PEG gel to obtain the St-tetra-PEG gels. Although the actual concentration of the introduced PNaAMPS in the St-tetra-PEG gels was not determined, it could be controlled by the NaAMPS monomer concentration in the precursor solutions for the molecular stent synthesis, *c*_NaAMPS_. Thus, we used *c*_NaAMPS_ as an index of the initial concentration of the molecular stent in the as-prepared St-tetra-PEG gels. The obtained St-tetra-PEG gels were first immersed in pure water or a 10^−4^ M NaCl aqueous solution.

**Figure 2:**
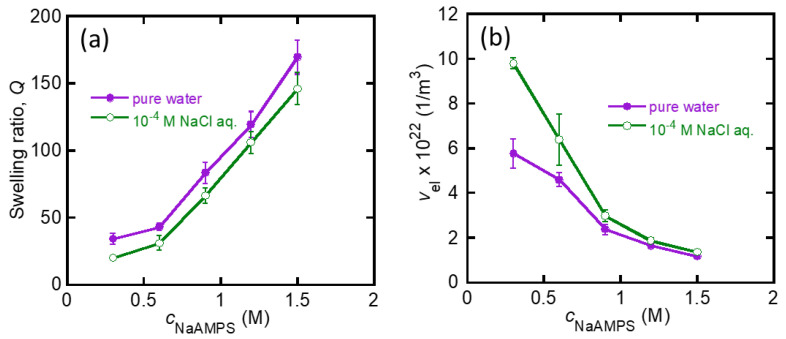
(**a**) Relationship between the swelling ratio, *Q*, and *c*_NaAMPS_ of the St-tetra-PEG gels in pure water or 10^−4^ M NaCl aqueous solution; (**b**) relationship between the number density of the elastically effective network strands, $\upsilon_{\mathrm{el}}$, and *c*_NaAMPS_ of the same St-tetra-PEG gels.

Figure 2a shows the equilibrium swelling ratio, *Q*, of the St-tetra-PEG gels in these solutions as a function of *c*_NaAMPS_. The *Q* of the St-tetra-PEG gels increased as the amount of molecular stent increased, reaching a maximum of approximately 170. This is because the concentration of mobile ions in the gels increases as the amount of molecular stent introduced increases, and thus $\Pi_{\mathrm{ion}}$ increases with *c*_NaAMPS_. The *Q* of the St-tetra-PEG gels in the NaCl solution is slightly lower than the corresponding *Q* in pure water because the introduction of mobile ions in the bath solution decreases the difference in the concentration of mobile ions inside and outside the gels. The $\upsilon_{\mathrm{el}}$ of the St-tetra-PEG gels was estimated by $\upsilon_{\mathrm{el}}=\upsilon_{\mathrm{el}0}/Q$, where $\upsilon_{\mathrm{el}0}$ is the $\upsilon_{\mathrm{el}}$ of the as-prepared tetra-PEG gel, as shown in Figure 2b. $\upsilon_{\mathrm{el}}$ decreases with *c*_NaAMPS_ because increases in *Q* with *c*_NaAMPS_ lead to the dilution of the network strands of the St-tetra-PEG gels.

**Figure 3:**
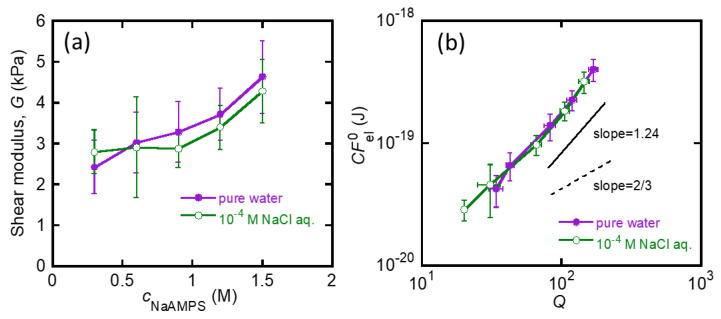
(**a**) Relationship between the shear modulus, *G*, and *c*_NaAMPS_ of the St-tetra-PEG gels swollen in pure water or 10^−4^ M NaCl aqueous solution; (**b**) relationship between $CF_{\mathrm{el}}^{0}$ and *Q* for the same St-tetra-PEG gels.

Figure 3a depicts the shear modulus, *G*, of the St-tetra-PEG gels in pure water or in 10^−4^ M NaCl aqueous solution as a function of *c*_NaAMPS_. For the St-tetra-PEG gels, *G* was in the order of several kPa and tended to increase with *c*_NaAMPS_ in this observation range. Subsequently, we calculated the $CF_{\mathrm{el}}^{0}$ of the St-tetra-PEG gels by dividing *G* by $\upsilon_{\mathrm{el}}$ and plotted this against *Q*, as shown in Figure 3b. The two series of data collapse into one master curve because the elasticity of the (St-)tetra-PEG gels should only depend on the network structure, which is directly controlled by the swelling ratio [26]. $CF_{\mathrm{el}}^{0}$ monotonically increased with *Q* owing to the swelling-induced stretching of the tetra-PEG network strands. Note that the empirical slope of the plots was 1.24, which is much larger than the theoretical value of 2/3 for a polymer network whose network strand obeys Gaussian statistics [19]. This large slope indicates that the elasticity of the highly swollen St-tetra-PEG gels no longer obeys the Gaussian model owing to the finite extensibility of the strands, as pointed out in a previous study [26].

### 2.2. Donnan Potential of the St-tetra-PEG Gels

**Figure 4:**
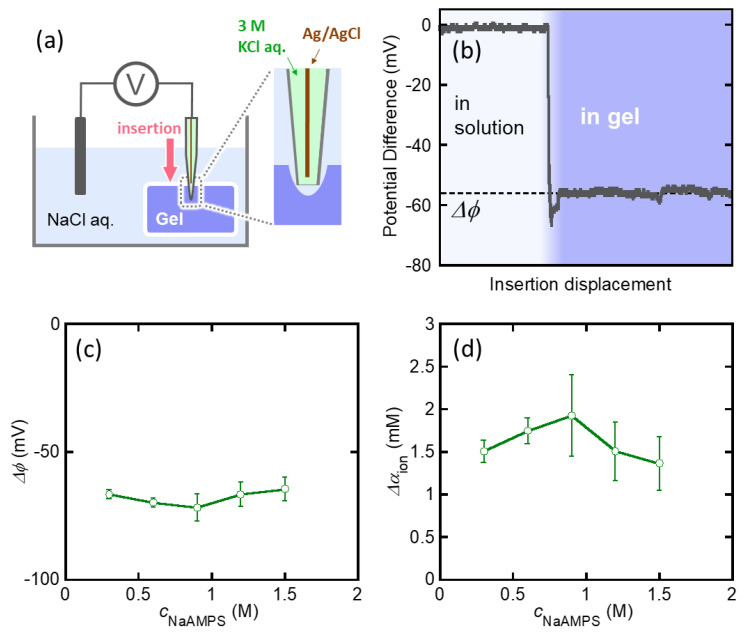
(**a**) Schematic illustration of how to perform the electric potential measurement; (**b**) a typical potential difference–insertion displacement curve; (**c**) effect of *c*_NaAMPS_ on Δϕ of the St-tetra-PEG gels in a 10^−4^ M NaCl aqueous solution; and (**d**) $\Delta \alpha_{\mathrm{ion}}$ of the St-tetra-PEG gels in a 10^−4^ M NaCl aqueous solution.

Figure 4a shows a schematic illustration of the microelectrode technique used to measure the electric potential difference between the gel and the solution. The reference electrode was immersed in a bath solution (10^−4^ M NaCl aq.), and a thin working electrode was inserted into the gel. Figure 4b shows a typical potential difference–insertion displacement curve. When the working electrode reached the gel bulk, the potential difference reached a plateau value, Δϕ. Under these measurement conditions, Δϕ was almost equal to the Donnan potential of the gel, $\phi_{D}$ [32]. Figure 4c demonstrates that Δϕ ($\approx \phi_{D}$) of the St-tetra-PEG gels equilibrated in 10^−4^ M NaCl aqueous solution as a function of *c*_NaAMPS_. The measured Δϕ is negative due to the anionic nature of PNaAMPS and is almost constant despite the variation in *c*_NaAMPS_. This independency of Δϕ from *c*_NaAMPS_ is because the *Q* of the St-tetra-PEG gels increases with *c*_NaAMPS_; therefore, the concentration of the molecular stent PNaAMPS in the swollen state becomes almost constant despite the difference in the initial PNaAMPS concentration.

$\Delta \alpha_{\mathrm{ion}}$ can be determined from the obtained Donnan potential, $\phi_{D},$ as follows. There are two monovalent mobile ions, Na^+^ and Cl^-^, in the system. At the swelling equilibrium, the Donnan equilibrium was established between the gel and bath solution. Thus, the Donnan potential of the gel is given by

(9) $$\phi_{D}=\frac{kT}{e}\ln\frac{\alpha_{{\mathrm{Na}}^{+}}^{env}}{\alpha_{{\mathrm{Na}}^{+}}^{g}}=\frac{kT}{e}\ln\frac{\alpha_{{\mathrm{Cl}}^{-}}^{g}}{\alpha_{{\mathrm{Cl}}^{-}}^{env}},$$

where *e* is the elementary charge [16]. From Equation (9), the activity of each mobile ion in the gel is given by

(10) $$\alpha_{{\mathrm{Na}}^{+}}^{g}=\alpha_{{\mathrm{Na}}^{+}}^{env}\exp\left(-\frac{e\phi_{D}}{kT}\right),$$

(11) $$\alpha_{{\mathrm{Cl}}^{-}}^{g}=\alpha_{{\mathrm{Cl}}^{-}}^{env}\exp\left(\frac{e\phi_{D}}{kT}\right).$$

According to the Debye–Hückel theory, if the ion concentration in a solution is sufficiently low, the ion activity is almost equal to the ion concentration:

(12) $$\alpha_{{\mathrm{Na}}^{+}}^{env}=\alpha_{{\mathrm{Cl}}^{-}}^{env}\approx c_{\mathrm{NaCl}}.$$

Thus, $\Delta \alpha_{\mathrm{ion}}$ is given by

(13) $${\Delta \alpha }_{\mathrm{ion}}=\left(\alpha_{{\mathrm{Na}}^{+}}^{g}+\alpha_{{\mathrm{Cl}}^{-}}^{g}\right)-\left(\alpha_{{\mathrm{Na}}^{+}}^{env}+\alpha_{{\mathrm{Cl}}^{-}}^{env}\right)\approx c_{\mathrm{NaCl}}\left(\exp\left(-\frac{e\phi_{D}}{kT}\right)+\exp\left(\frac{e\phi_{D}}{kT}\right)-2\right).$$

The calculated $\Delta \alpha_{\mathrm{ion}}$ of the St-tetra-PEG gels equilibrated in a 10^−4^ M NaCl aqueous solution is shown in Figure 4d. The corresponding $\Pi_{\mathrm{ion}}$ is then given by

(14) $$\Pi_{\mathrm{ion}}=\Delta \alpha_{\mathrm{ion}}kT\approx kTc_{\mathrm{NaCl}}\left(\exp\left(-\frac{e\phi_{D}}{kT}\right)+\exp\left(\frac{e\phi_{D}}{kT}\right)-2\right).$$

Finally, $\frac{\Delta \alpha_{\mathrm{ion}}}{\upsilon_{\mathrm{el}}}kT$, which is on the right side of Equation (8), can be calculated from the measured $\phi_{D}$ as

(15) $$\frac{\Delta \alpha_{\mathrm{ion}}}{\upsilon_{\mathrm{el}}}kT\approx \frac{kTc_{\mathrm{NaCl}}}{\upsilon_{\mathrm{el}}}\left(\exp\left(-\frac{e\phi_{D}}{kT}\right)+\exp\left(\frac{e\phi_{D}}{kT}\right)-2\right).$$

### 2.3. Balance between Elasticity and Ionic Osmosis

**Figure 5:**
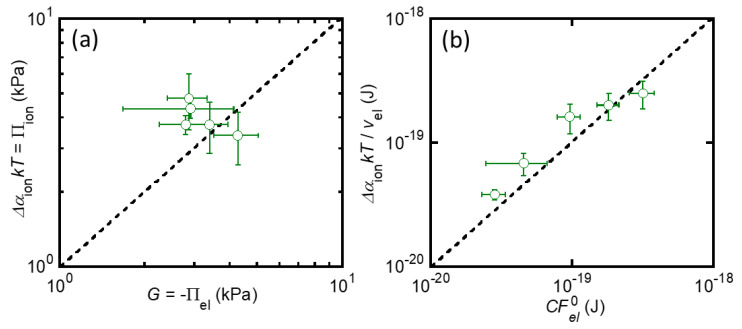
Relationships between elastic pressure and the ionic osmotic pressure of the St-tetra-PEG gels; (a) *G* vs.$\;\Delta \alpha_{\mathrm{ion}}kT$ (macro scale) and (b) $CF_{\mathrm{el}}^{0}$ vs. $\frac{\Delta \alpha_{\mathrm{ion}}}{\upsilon_{\mathrm{el}}}kT$ (single strand scale). Note that the $\Delta \alpha_{\mathrm{ion}}$ was experimentally determined through the potential measurement.

Now, we can directly validate the two equations that describe the equilibrium swelling condition of strongly charged gels, Equations (6) and (8), because both sides of these equations have been experimentally determined without any fitting parameters. First, the validity of Equation (6), which shows the balance of elastic energy and ionic osmotic pressure at the macroscale, was investigated. Figure 5a shows the relationship between *G* ($=-\Pi_{\mathrm{el}}$) and $\Delta \alpha_{\mathrm{ion}}kT\left(\approx \Pi_{\mathrm{ion}}\right)$ of the St-tetra-PEG gels immersed in a 10^−4^ M NaCl aqueous solution. The dashed line in the graph represents $G=\Delta \alpha_{\mathrm{ion}}kT$. All the data points are close enough to the dashed line, suggesting that Equation (6) works with sufficient accuracy in the St-tetra-PEG gel system. Then, the validity of Equation (8), which shows the balance between elasticity and ionic osmosis at the single-strand level, was investigated. Figure 5b shows the relationship between $CF_{\mathrm{el}}^{0}$ and $\frac{\Delta \alpha_{\mathrm{ion}}}{\upsilon_{\mathrm{el}}}kT$ of the St-tetra-PEG gels. As shown in Figure 5a, all data points were located near the dashed line corresponding to the balance between elasticity and ionic osmosis. This indicates that Equation (8) is valid for this system. Thus, in this study, we succeeded in clarifying the balance between the elasticity and ionic osmosis of the St-tetra-PEG gels immersed in 10^−4^ M NaCl solution, at both the macro-and single-strand levels, by the experimental determination of both effects.

### 2.4. St-tetra-PEG Gels in a Strong Ionic Environment

**Figure 6:**
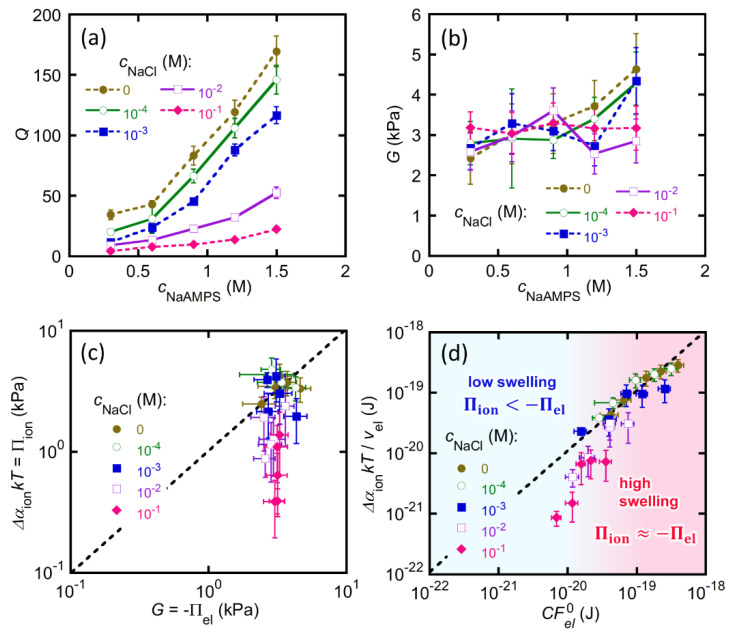
(**a**) Swelling ratio and (**b**) shear modulus of the St-tetra-PEG gels with varied $c_{\mathrm{NaAMPS}}$ depending on $c_{\mathrm{NaCl}}$; (**c**) *G* vs.$\;\Delta \alpha_{\mathrm{ion}}kT;$ and (**d**) $CF_{\mathrm{el}}^{0}$ vs. $\frac{\Delta \alpha_{\mathrm{ion}}}{\upsilon_{\mathrm{el}}}kT$ of the St-tetra-PEG gels depending on $c_{\mathrm{NaCl}}$. Note that $\Delta \alpha_{\mathrm{ion}}$ was not directly measured but estimated with Equations (16) and (17).

The validity of the measured Donnan potentials of the St-tetra-PEG gels should be further confirmed. Thus, we performed another set of experiments, which involved the immersion of the St-tetra-PEG gels with various $c_{\mathrm{NaAMPS}}$ in NaCl aqueous solutions of higher concentrations. Figure 6a,b show the *Q* and *G* of the series of St-tetra-PEG gels swollen in NaCl aqueous solutions with varied $c_{\mathrm{NaCl}}$. For the swelling ratio, *Q* remarkably decreased with increasing $c_{\mathrm{NaCl}}$, suggesting that the ionic osmotic pressure of the St-tetra-PEG gels was effectively suppressed by the large mobile ion concentration of the bath solutions.

As discussed in a previous report, when $c_{\mathrm{NaCl}}$ is too high, the Donnan potential inside the gel cannot be determined precisely using our microelectrode technique [32]. As illustrated in Figure 4a, a microcrack is formed ahead of the inserted working electrode. If the ionic strength is sufficiently low, the effective measurement range ahead of the microelectrode is very large because of the large Debye length; therefore, the measured Δϕ certainly reflects the bulk properties of the gels. However, if the ionic strength is large, the effective measurement range is so small that the measured Δϕ is strongly affected by the presence of microcracks. As a result, when $c_{\mathrm{NaCl}}$ is too high, the absolute values of the measured Δϕ of the St-tetra-PEG gels were smaller than the actual Donnan potential $\phi_{D}$. Therefore, we calculated $\Delta \alpha_{\mathrm{ion}}$ of the gels with large $c_{\mathrm{NaCl}}$ from the Donnan potential of the gels in 10^−4^ M NaCl aq. with several assumptions. Considering the electroneutrality in the gel and the Donnan equilibrium, one can derive the following equation under the assumptions of a quasi-infinite volume of the bath solution and low concentration of mobile ions both in the gel and the bath solution [17]:

(16) $$\Pi_{\mathrm{ion}}=\Delta \alpha_{\mathrm{ion}}kT≅kT\left(\sqrt{\gamma c_{p^{-}}^{g}{}^{2}+4c_{\mathrm{NaCl}}{}^{2}}-2c_{\mathrm{NaCl}}\right),$$

where $c_{p^{-}}^{g}$ is the concentration of the fixed negative charges on the molecular stent in the St-tetra-PEG gel and *γ* is the ratio of mobile counterions among the total counterions in the gel. When $c_{\mathrm{NaCl}}$ is 10^−4^ M, the $\Pi_{\mathrm{ion}}$ of the St-tetra-PEG gel with certain $c_{\mathrm{NaAMPS}}$ can be determined from the Donnan potential using Equation (14). Here, we additionally assume that *γ* is independent of $c_{\mathrm{NaCl}}$, as in previous studies [16,21,32]. As $c_{p^{-}}^{g}$ should be inversely proportional to the swelling ratio *Q*, the $\gamma c_{p^{-}}^{g}$ of the St-tetra-PEG gel in the bath solution of any $c_{\mathrm{NaCl}}$ can be calculated by the following equation:

(17) $$\gamma c_{p^{-}}^{g}≅\gamma c_{p^{-}}^{g}\;\mathrm{at}\;c_{\mathrm{NaCl}}:\;10^{-4}\;M\times \frac{Q\;\mathrm{at}\;c_{\mathrm{NaCl}}:\;10^{-4}\;M}{Q}.$$

By using the $\gamma c_{p^{-}}^{g}$ estimated using Equation (17), the $\Pi_{\mathrm{ion}}$ of the St-tetra-PEG gels with large $c_{\mathrm{NaCl}}$ can be obtained by applying Equation (16) without the separate determination of *γ* and $c_{p^{-}}^{g}$.

Figure 6c shows the balance between the elastic pressure and ionic osmotic pressure of the St-tetra-PEG gels in NaCl solutions of various $c_{\mathrm{NaCl}}$ concentrations. When $c_{\mathrm{NaCl}}$ was 10^−3^ M or lower, where the gels are highly swollen, the estimated ionic osmotic pressure was well balanced with the elastic pressure. This result implies that the estimated ionic osmotic pressure of the St-tetra-PEG gels with $c_{\mathrm{NaCl}}$ = 10^−4^ M is reliable. On the other hand, when $c_{\mathrm{NaCl}}$ was 10^−2^ M, some data points shifted downward from the dashed line. Moreover, when $c_{\mathrm{NaCl}}$ was 10^−1^ M, all data points were located far from the dashed line. These shifts indicate that the estimated ionic osmotic pressure was remarkably lower than the elastic pressure. The deviation of ionic osmosis from elasticity becomes clear, as shown in Figure 6d, which illustrates the balance between elasticity and ionic osmosis at the single-strand level. When $CF_{\mathrm{el}}^{0}$ (elastic energy per strand) is sufficiently large, elasticity and ionic osmosis are balanced. However, when $CF_{\mathrm{el}}^{0}$ is relatively small, the measured $\frac{\Delta \alpha_{\mathrm{ion}}}{\upsilon_{\mathrm{el}}}kT$ is smaller than $CF_{\mathrm{el}}^{0}$, and the relative difference between the two increases as $CF_{\mathrm{el}}^{0}$ decreases. The deviation of ionic osmosis from elasticity appears around $\frac{\Delta \alpha_{\mathrm{ion}}}{\upsilon_{\mathrm{el}}}kT$ = 4 ×10^−20^ J, corresponding to ~10 *kT* per strand. Thus, one can roughly assume that:

(18) $$\{\begin{array}{c} \Pi_{\mathrm{ion}}<-\Pi_{\mathrm{el}},\;\;\frac{\Delta \alpha_{\mathrm{ion}}}{\upsilon_{\mathrm{el}}}<10\\ \Pi_{\mathrm{ion}}≅-\Pi_{\mathrm{el}},\;\;\frac{\Delta \alpha_{\mathrm{ion}}}{\upsilon_{\mathrm{el}}}\geq 10 \end{array}.$$

The first regime, where $\Pi_{\mathrm{ion}}<-\Pi_{\mathrm{el}}$, can be called a low-swelling regime. In this regime, the ionic osmotic pressure of the St-tetra-PEG gels is small because of either the high ionic environment of the bath solution or the low initial concentration of the molecular stent. As a result, the equilibrium swelling ratio, *Q*, of the St-tetra-PEG gels is not large, and the polymer volume fraction, ϕ, is non-negligible. Thus, the contribution of the osmotic pressure of mixing, $\Pi_{\mathrm{mix}}\left(\phi \right),$ to the total swelling pressure of the gels must be considered. The second regime where $\Pi_{\mathrm{ion}}≅-\Pi_{\mathrm{el}}$ can be called the high-swelling regime. In this regime, because the $\Pi_{\mathrm{ion}}$ of the St-tetra-PEG gels is sufficiently large, the gels swell in the bath solution. The resulting ϕ of the swollen St-tetra-PEG gels is so small that the contribution of $\Pi_{\mathrm{mix}}$ to the total swelling pressure is negligible, and Equations (6) and (8) are valid for this regime. One active mobile ion in the gel adds a free energy of *kT*, and the average contour length of the network strand of the St-tetra-PEG gel used in this study was 82 nm [26]. Thus, for this system, the requirement for a high-swelling regime $\left(\frac{\Delta \alpha_{\mathrm{ion}}}{\upsilon_{\mathrm{el}}}\geq 10\right)$ corresponds to the situation where one excess active mobile ion exists per 8 nm or less of the network strands in the gel. These values are the threshold conditions that divide the low-and high-swelling regimes of the St-tetra-PEG gels. The authors believe that these values vary depending on the system and may be affected by several conditions, such as the average contour length of the network strands and initial polymer volume fraction. The effects of the preparation conditions of charged gels on these threshold values should be investigated further.

Finally, we should mention the limitations of the applicability of our findings. In this study, we used semi-interpenetrating gels consisting of a non-ionic network and a linear polyelectrolyte and experimentally confirmed the balance of $\Pi_{\mathrm{ion}}$ and $\Pi_{\mathrm{el}}$. However, it is not clear whether the same results can be obtained for a typical polyelectrolyte gel (ionic network with fixed charges). If the network itself has fixed charges, other contributions, such as electrostatic interactions between the fixed charges, should also affect the swelling pressure. Thus, the applicability of our results to general polyelectrolyte gels should be confirmed in future studies.

## 3. Conclusions

In this study, we experimentally verified Equations (6) and (8), which describe the balance between elasticity and the ionic osmosis of the highly swollen charged gels at both the macroscale and single-strand scales. The conditions necessary for applying these equations were also analyzed. Both the elastic and ionic osmotic pressures were directly determined experimentally using the indentation test and the microelectrode technique. Using tetra-PEG gels with the molecular stent method enables analysis at the single-strand level. This fully experimental study on the swelling of charged gels will further understanding of their swelling behavior and lead to improvement in their swelling theories.

## 4. Materials and Methods 

### 4.1. Materials

Amine-terminated 4-arm polyethylene glycol SUNBRIGHT PTE-200PA (TAPEG) and NHS-terminated 4-arm polyethylene glycol SUNBRIGHT PTE-200HS (TNPEG) were purchased from NOF Corporation; Tokyo, Japan and used as received. The average molecular weight of each pre-polymer was 20 k. 2-Acrylamido-2-methylpropane sulfonic acid sodium salt (NaAMPS: Toa Gosei Co. Ltd.; Tokyo, Japan), 2-oxogultaric acid (FUJIFILM Wako Pure Chemical Industries, Ltd.; Osaka, Japan), and sodium chloride (NaCl: Wako Pure Chemical Industries, Ltd.) were used as received.

### 4.2. Preparation of the St-tetra-PEG Gels

St-tetra-PEG gels were synthesized as previously reported [26]. Briefly, TAPEG and TNPEG were dissolved in phosphate buffer (pH 7.4) and citrate–phosphate buffer (pH 5.8), respectively, at a concentration of 40 mg/mL, which is equal to their overlapping concentration. Equal amounts of each pre-polymer solution were mixed and injected into rectangular glass molds using a plastic syringe throughout the filter membrane. The tetra-PEG gels were obtained by leaving the molds filled with solutions for at least 12 h at room temperature. The as-prepared tetra-PEG gels were used as the reference state. The tetra-PEG gels were then immersed into the aqueous solution of NaAMPS with concentrations, *c*_NaAMPS_, of 0.3–1.5 M and 2-oxoglutaric acid with a concentration of 0.1 mol% (relative to the monomer) for at least 24 h. The gels were then sandwiched between two glass plates and irradiated with 365 nm UV light (intensity: 4 mW cm^−2^) for at least 3 h to synthesize PNaAMPS as a linear polyelectrolyte (molecular stent) in the tetra-PEG gels. The obtained tetra-PEG gels containing linear PNaAMPS were called St-tetra-PEG gels. The St-tetra-PEG gels were immersed in pure water or an aqueous solution of 10^−4^–10^0^ M NaCl for 1 week until swelling equilibrium was reached. The swelling ratio of the equilibrated St-tetra-PEG gels was determined by

(19) $$Q=\frac{V}{V_{0}},$$

where *V* and *V*_0_ are the volumes of the gel at the state of interest and at the reference state, respectively.

### 4.3. Indentation Test

The moduli of the St-tetra-PEG gels were measured by an indentation test using a universal mechanical testing device (Tensilon RTC-1150A: Orientec Co.; Tokyo, Japan) equipped with a hemispherical steel indenter, as previously reported [26]. The shear modulus of the St-tetra-PEG gels was obtained based on Hertz’s contact theory as follows:

(20) $$G=\frac{3}{16}l^{-\frac{3}{2}}fR^{-\frac{1}{2}},$$

where *G*, *l*, *f*, and *R* are the shear modulus, displacement of the indenter, force, and radius of the indenter (2 mm), respectively [26]. It should be noted that we assume that the Poisson’s ratio of the gels is 0.5 for the derivation of Equation (20). The Poisson’s ratio of the gels depends on their deformation rates. It is very close to 0.5 if the deformation rate is sufficiently large but deviates from 0.5 if the deformation rate is small due to solvent migration [45]. In this experiment, the indentation velocity was set to 1 mm/min, the solvent migration was negligible and the Poisson’s ratio of the gel was assumed to be 0.5 [26,46]. *G* was determined from the slope of the linear regime of the *f*–$l^{\frac{3}{2}}$ curve obtained at a small displacement (0–0.1 mm) based on Equation (20). The values of *G* are the average of at least four measurements. All the experiments were performed at room temperature.

### 4.4. Electric Potential Measurement

The electric potential difference between the St-tetra-PEG gels equilibrated in a 10^−4^ M NaCl aqueous solution and the bath solution was measured by the microelectrode technique, as shown in Figure 4a [32]. A carbon electrode was used as the reference electrode. For the working electrode, thin glass capillary tubes (tip diameter: <1 µm) were first prepared by pulling the original glass capillary tube (outer diameter 1.0 mm, inner diameter 0.78 mm, Sutter Instrument Co.; Novato, CA, USA) using a puller (P-1000, Sutter Instrument Co.). A 3 M KCl solution was added to the glass capillary and a reversible silver/silver chloride electrode (Ag/AgCl) was inserted into it to prepare the working microelectrode. Both electrodes were connected to an oscilloscope (DS-4264, Iwatsu electric Co, Ltd.; Tokyo, Japan) via a high-impedance intracellular preamplifier (Model 8700 Cell Explorer, Dagan Co.; Minneapolis, MN, USA) to determine the potential difference. The reference electrode was immersed in the bath solution, and the working electrode was inserted into the gel at a constant speed of 395 nm/s using a micromanipulator (DMA-1511, Narishige Scientific Instrument Lab.; Tokyo, Japan) to obtain the potential difference–insertion displacement curve. A potential difference plateau is typically observed when the working electrode reaches the bulk gel. The average potential difference between the gel and bath solution, Δϕ, was determined by taking the average of the potential differences in this plateau region. The values of Δϕ are the averages of at least three measurements. All the measurements were performed at room temperature.

## Data Availability

The data presented in this study are available on request from the corresponding author.
